# Endoscopic far-lateral supracerebellar infratentorial approach for resection of clival chordoma: case report

**DOI:** 10.3389/fonc.2024.1448063

**Published:** 2024-10-25

**Authors:** Song Han, Yang Bai, Xiaoyu Sun, Ligang Chen, Yang Gao, Hongzhe Liu, Huanhuan Li, Jieyu Lai, Sizhe Feng

**Affiliations:** Department of Neurosurgery, General Hospital of Northern Theater Command, Shenyang, Liaoning, China

**Keywords:** clival chordoma, supracerebellar infratentorial approach, endoscope, surgical approach, case report

## Abstract

**Introduction:**

The surgery of clival chordoma remains one of the most formidable challenges for neurosurgeons because of its location at great depth in the cranium and proximity to critical neurovascular structures. Here, we describe the technique and feasibility of the purely endoscopic far-lateral supracerebellar infratentorial approach (EF-SCITA) for resection of an intradural clival chordoma.

**Case description:**

A 68-year-old women presented with sudden ptosis on the left side for two weeks. Imaging examinations revealed an upper-middle clival lesion that transgressed dural confines towards the posterior fossa, which was separated from the sphenoid cavity by an intact thin layer of membrane structure in front. For surgery, the EF-SCITA approach via suboccipital craniotomy was attempted for protecting surrounding neurovascular tissue and the membrane barrier under direct vision. The patients were placed in a “head-up” lateral park-bench position. With the endoscopic holder, endoscopic procedures were performed using standard two-hand microsurgical techniques by one surgeon. Tentorium incision allowed a working corridor toward the clival bulge through the crural cistern, without brain traction seen in traditional retrosigmoid approach. Efficient tumor debulking facilitated the exposure of surrounding critical structures, including ipsilateral CN III and superior cerebellar artery above, the brainstem and basilar artery posteriorly, as well as ipsilateral CN VI displaced laterally, and subsequent tumor separation from them. Step-wise tumor resection was performed within dural and bone confines. After significant tumor removal, the pituitary stalk could be visualized anteriorly, together with contralateral internal carotid artery and CN III. Postoperative MRI depicted gross total excision of the lesion. The patient on follow-up at one year had complete recovery of cranial nerve functions, without signs of cerebrospinal fluid rhinorrhea.

**Discussion:**

This technique combines advantages of the posterolateral approach and endoscopy, allowing access to the upper-middle clivus with seemingly low risks of postoperative morbidity. It would be a safe and effective alternative for resection of this rare entity.

## 1 Introduction

Clival chordomas are rare midline malignancies that locally invade and metastasize, with a high recurrence rate (1). They are usually extradural masses originating within the bone, and intradural chordomas are rare (2). For treatment, special emphasis is put on preservation of neurological function, typified by a general paradigm of maximally safe cytoreductive surgery and advanced radiation delivery techniques (3). Surgical excision of clival lesions presents neurosurgeons with a formidable challenge since it is deeply nested in the central of cranial base and surrounded by critical neurovascular structures, such as abducens nerve, basilar artery (BA), and brainstem. The complications of surgery for this entity range from none to severe disabling neurological deficits. A variety of surgical approaches, including midline and lateral routes, have been described to access clival lesions (4).

Herein, we report a case of endoscopic far-lateral supracerebellar infratentorial approach (EF-SCITA) in the removal of clival chordoma, with specific emphasis on surgical nuances. The advantages of the approach and its potential indications in the treatment of chordomas are analyzed. In the discussion, we briefly summarize breakthroughs and insights on operative approaches for clival chordoma.

## 2 Case description

**Figure 1:**
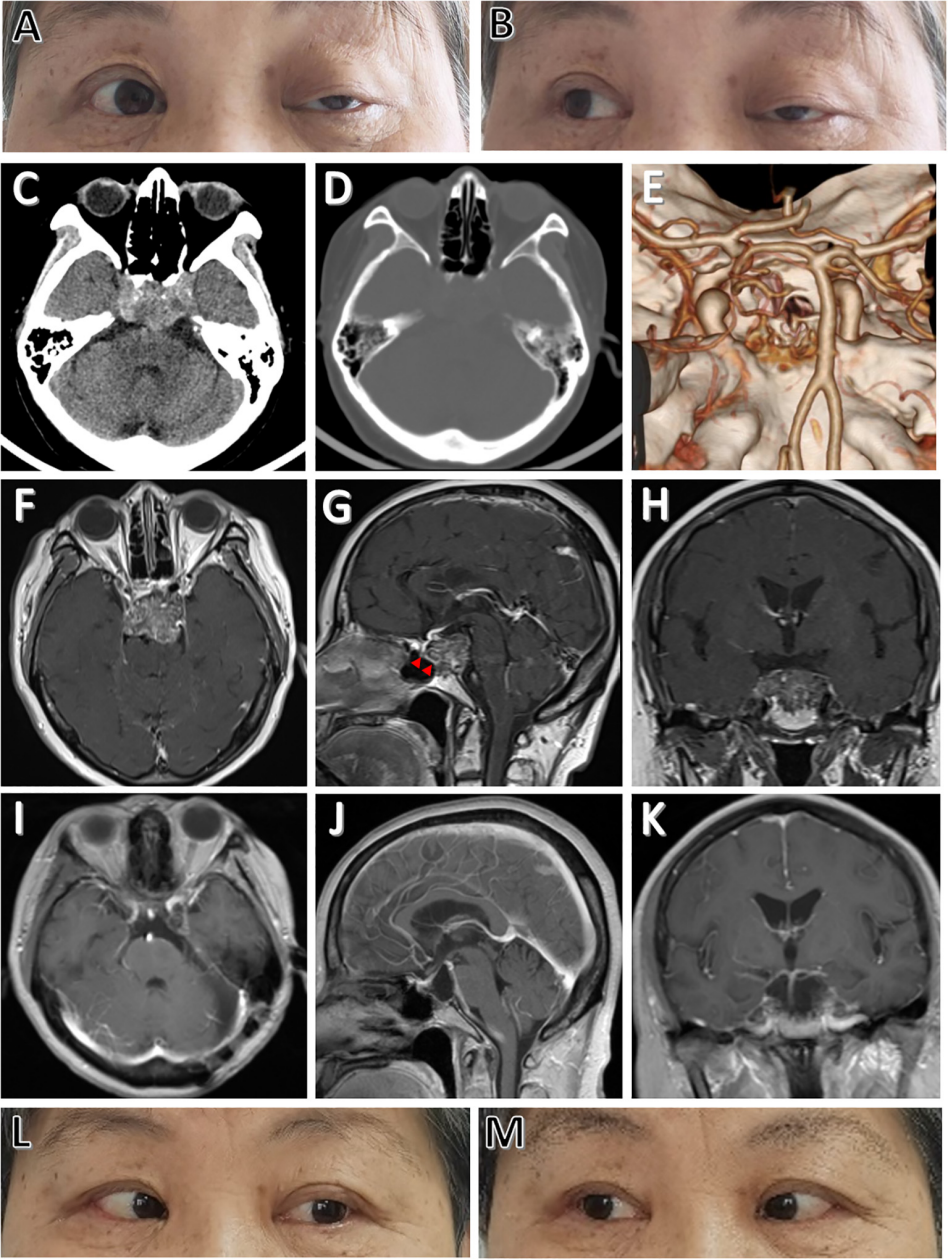
Physical and radiological evaluations of the patient preoperatively and postoperatively. **(A, B)** Preoperative physical examinations of the patient showing cranial nerve paralysis of left CN III and CN VI. **(C)** Preoperative CT image showing a hyperdense lesion located at the upper clivus. **(D, E)** Preoperative CT and CT angiography images showing clival bone destruction. **(F-H)** Preoperative post-contrast (gadolinium-enhanced) axial **(F)**, sagittal **(G)**, and coronal **(H)** MRI images showing thick enhancement of the lesion. **(I-K)** Postoperative post-contrast axial **(I)**, sagittal **(J)**, and coronal **(K)** MRI scans performed one year after surgery showing total removal of the lesion. **(L, M)** Postoperative physical examinations showing complete functional recovery of CN III and CN VI paralysis at one-year follow-up. The red arrowheads indicate the intact thin layer of membrane structure separating the tumor from the sphenoid cavity.

A 68-year-old female presented with sudden ptosis on the left side for two weeks. Neurological exams indicated left 3^rd^ and 6^th^ nerve paralysis, and no other neurological deficit was found (**Figures 1A, B**). Routine laboratory investigations, including endocrinological studies, were normal. No specific past, medical, family and psycho-social history was reported. Computed tomography scans revealed lytic lesion of the upper and middle clivus (**Figures 1C–E**). Magnetic resonance imaging (MRI) depicted a medium-sized clival mass with intradural extension and pontine compression (**Figures 1F–H**). These clinico-radiological findings are most consistent with a clival chordoma. It is worth noting that there is a thin layer of membrane structure remaining intact, separating the lesion from the sphenoid cavity in front (**Figure 1G**). Midline approaches would damage this barrier and increase the risk of postoperative cerebrospinal fluid (CSF) leak. In this case, the EF-SCITA approach was attempted for tumor resection (Video 1)^1^ ^,^ ^2^. We speculated that this posterolateral route could better protect surrounding neurovascular structures under direct vision and provide the possibility for preserving the natural barrier in this special case.

**Figure 2:**
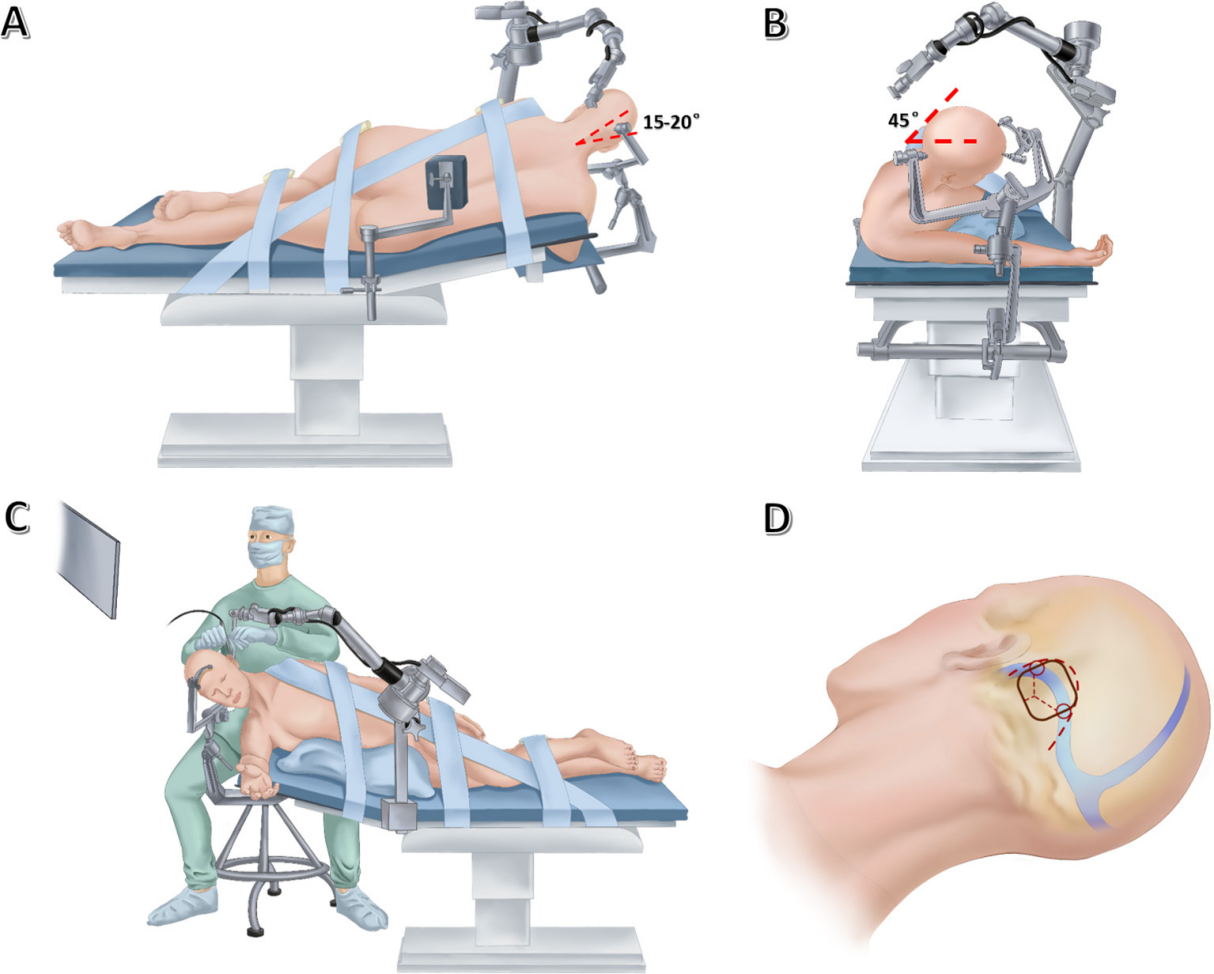
Drawing showing the lateral **(A)** and anterior **(B)** views of patient’s position, the lateral view of neurosurgeon’s position **(C)**, as well as skin incision (thick dashed line), craniotomy (black solid line) and dural incision (thin dashed lines) **(D)** in endoscopic far-lateral supracerebellar infratentorial approach for clival chordoma resection.

Surgery is performed in a right modified lateral park-bench position. The operating table is adjusted to a reverse Trendelenburg position at an angle of 10° to allow better venous drainage. Then, the head is placed in upper flexion of 15-20° to further allow gravity retraction of the cerebellum (**Figure 2A**) and rotated 45° upward to keep the sagittal suture parallel to the floor (**Figure 2B**). The surgeon sits behind the upper shoulder at an angle of 30-45° to the longitudinal axis of the bed. The left shoulder of the patient is pulled in the ventral and caudal direction as far as possible, thus facilitating unlimited room for the operator’s hands. The inclined back of the patient supports the left hand of the surgeon for reducing fatigue (**Figure 2C**). For the fixation of head, the Mayfield skull clamp was used. The two pins are located at the frontal bone, while the head rests on the 1-pin rocker arm in which the pin is at the contralateral parietal bone near the inion. This configuration is intended to minimize obstruction of the operative field (**Figures 2A, B**).

Currently, both static and freehand dynamic endoscopy techniques have been described in fully endoscopic surgery for posterior fossa lesions (5, 6). With a dynamic endoscopic technique, subtle movements of the lens could enhance depth perception and spatial awareness without the need for stereoscopic three-dimensional vision. But frequent insertion and extraction of the lens may damage vital neurovascular structures during surgery. Considering this, an endoscopic arm holder was implemented here for offering protection against damaging neural and vascular structures behind the camera. The static endoscopic configuration further enables the surgeon to sit behind the patients for operations and alleviates the workload of the assistant. In this case, the pneumatic arm holder (Karl Storz, Culver City, CA) is placed on the contralateral bedside, allowing it to arch over the patient’s head. The holding arm secures the endoscope in position, allowing bimanual operations. The monitor is placed in a direct line-of-sight from where the primary surgeon sits (**Figures 2A–C**).

**Figure 3:**
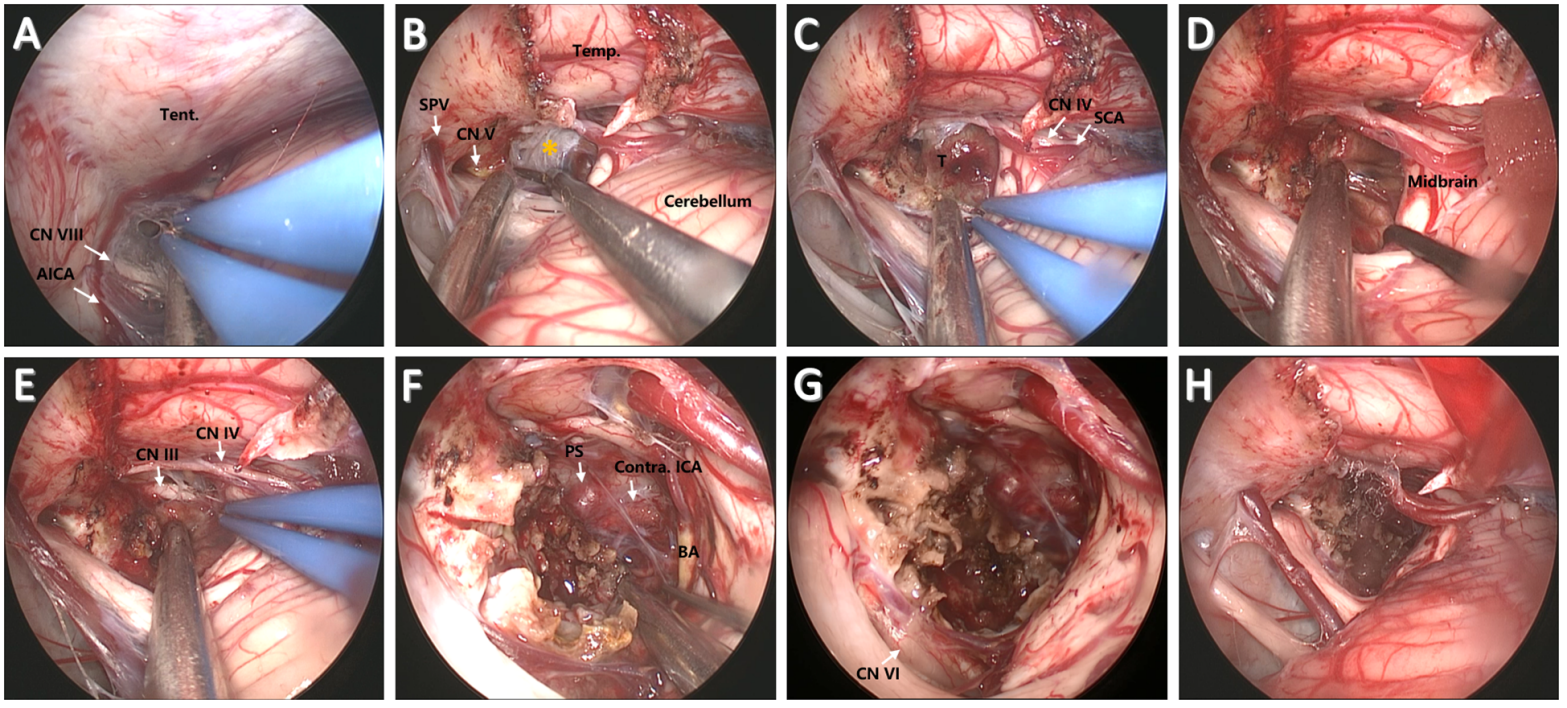
Surgical procedure and nuances of the EF-SCITA approach for resection of clival chordoma. **(A)** Endoscopic explorations in the lateral supracerebellar infratentorial space. **(B)** Incision of the tentorium cerebelli for entering into the crural cistern. **(C)** Tumor exposure after arachnoidal dissection in the crural cistern. **(D)** Tumor separation from the brainstem posteriorly after debulking. **(E)** Tumor separation from ipsilateral CN III upwardly. **(F)** The exposure of PS, BA, and contralateral ICA after radical tumor removal. **(G)** The exposure of ipsilateral CN IV after total tumor removal. **(H)** The surgical field after tumor removal. AICA, anterior inferior cerebellar artery; BA, basilar artery; CN III, oculomotor nerve; CN IV, trochlear nerve; CN V, trigeminal nerve; ICA, internal carotid artery; Temp., temporal lobe; PS, pituitary stalk; SPV, superior petrosal vein; SCA, superior cerebellar artery; T, tumor; Tent., tentorium cerebelli. The yellow asterisks indicate arachnoidal membrane in the crural cistern.

For suboccipital craniotomy, a reversed U-shaped retroauricular incision (Dandy incision) is performed. Then, the musculocutaneous flap is turned downwards to expose the suboccipital region. The curvilinear incision allows for a larger bone flap that facilitates subsequent instrument insertion and surgical operations compared with the linear incision. Most importantly, this mobilization of the flap is out of the way of the endoscope and surgical instruments. In addition, Dandy incision avoids deep muscle dissection, thus minimizing the risk of postoperative neuropathic pain (7). Then, the entire transverse sinus and transverse-sigmoid junction are revealed through suboccipital craniotomy (3.5 cm × 3.5 cm), and a T-shaped dural flap is created (**Figure 2D**). Intraoperative CSF drainage from the lateral cerebellomedullary cistern or lumbar cistern allows the cerebellum to spontaneously retract from the petrous bone and tentorium cerebelli. Favorable cerebellar relaxation creates a wide corridor toward the CPA region through the supracerebellar space (**Figure 3A**).

First, the tentorium cerebelli was opened, during which much attention should be paid to the protection of CN IV (**Figure 3B**). The clival bulge that protruded into the prepontine and interpeduncular cisterns could be identified after arachnoidal dissection (**Figure 3C**). Then, a small transverse incision was taken on the bulge. The content mixed with blood component could be easily removed. After initial debulking, the capsule of the tumor is then gently dissected off the brainstem and BA posteriorly (**Figure 3D**). Next, the tumor was carefully separated from the CN III and superior cerebellar artery (SCA) above (**Figure 3E**). Tumor resection was then continued in the available surgical field. The soft tumor component with interspersed bone pieces was removed using fine microneurosurgical techniques. After significant tumor debulking, the pituitary stalk could be visualized anteriorly, together with contralateral internal carotid artery (ICA) and CN III (**Figure 3F**). In addition, ipsilateral CN VI that was displaced laterally (**Figure 3G**) could be observed at the site of its clival dural entry site and its initial course in relationship with the tumor after radical tumor removal (**Figure 3H**). Thus, tumor resection should be conducted within the dural confines to protect CN VI. Along with radical tumor resection, special attention should be paid to the protection of the remnant layer of membrane structure in front of the tumor.

The postoperative course was uneventful, with no complications such as CSF leak. The patient was discharged on 7^th^ postoperative day. Postoperative MRI depicted gross total resection (**Figures 1I–K**). Histopathological examinations demonstrated findings consistent with a chordoma and the patient was recommended for standard radiation treatment. The patient on follow-up at 1 year had complete recovery of cranial nerve functions (**Figures 1L, M**).

## 3 Discussion

### 3.1 Current surgical strategies for clival chordoma

**Figure 4:**
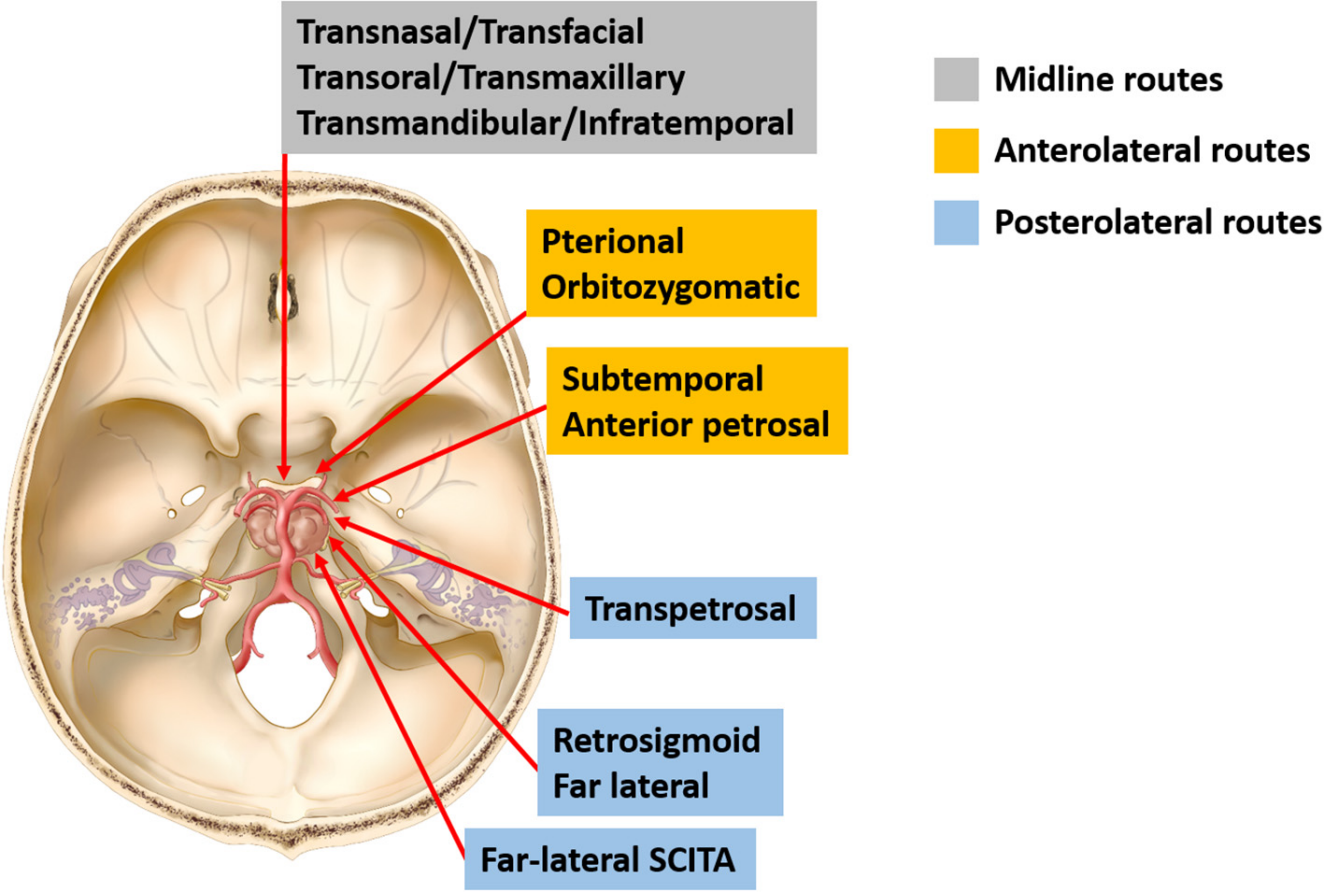
Schematic diagram of surgical approaches for resection of clival chordomas. Current approaches for resection of clival chordomas could be specified as midline, anterolateral, and posterolateral approaches. SCITA, supracerebellar infratentorial approach.

The principle of clival chordoma surgery is maximally safe cytoreductive surgery while minimizing neurological dysfunctions (3). According to surgical directions accessing the clivus, available approaches could be classified into midline (or anterior) and lateral (or transcranial) routes (**Figure 4**). Traditionally, multiple transcranial approaches have been implemented to achieve radical tumor removal with acceptable risks. Many of them represent variations of lateral operative corridors with modifications in bone resection, which involve extensive drilling of the skull base or significant brain retraction followed by dissection and maneuvering through critical neurovascular structures to reach the tumor (4, 8). Generally speaking, the upper clivus is better approached through an orbitozygomatic approach; the midclivus could be accessed from the transpetrosal route (anterior, posterior, or total petrosectomy); and the lower clivus is usually reached by a far-lateral approach (1, 4).

With advances in modern endoscopy, the last two decades have witnessed a paradigm shift in the domain of skull base chordoma from open transcranial approaches to extended endoscopic endonasal approaches (EEEA). The philosophy of this technique lies in minimizing the opening at the surface while maximizing resection closer to the target. The trans-sellar, sphenoidal and infrasphenoidal corridors can be used alone or in combination for effective resection of the lesion. The sphenoidal corridor provides excellent access to a mid-clival tumor, which relies on a well-pneumatized sphenoid sinus. The trans-sellar route is used for clival chordomas located in the retrosellar space, which entails complicated procedures including pituitary transposition and the resection of dorsum sellae. Thus, much attention should be paid to postoperative pituitary hypofunciton. For the infrasphenoidal route, a trans-oral approach could be added to better identify the foramen magnum and hypoglossal canals (1, 9).

Thus, for clival lesions with their bulk in the midline and having a major extension in the sphenoid sinus, the EEEA is the most appropriate approach in terms of avoiding neurovascular injuries. As the pathology starts spreading laterally, the endoscopic transclival approach can be augmented in the coronal plane by the addition of working around the ICA. When the tumor location is too lateral or inferior, an open approach or a combination of endoscopic and open approaches in stages should be considered (9).

Apart from the transnasal approach, other midline routes to the clivus include transfacial, transmaxillary, transoral, transmandibular and infratemporal approaches with related morbidity in terms of cosmesis and function (10). Despite variations in prior literature, midline approaches have been advocated as yielding superior outcomes. However, recent analysis failed to identify any discernable disparity in rates of gross total resection, recurrence, or mortality between midline and lateral approaches. The only finding of significance was that CSF leakage was higher in midline approaches while lateral surgical approaches showed a predilection for cranial nerve palsies (4, 8).

### 3.2 EF-SCITA for clival lesions

As a distinct variant of the SCITA approach, far-lateral SCITA was firstly described by Spetzler et al. in 2000 to access the posterolateral midbrain (11). Later, it proved its versatility and clinical practicality in treating tumors residing in centrally-located intra-axial structures like the splenium, brainstem, mesial temporal lobe, as well as skull base extra-axial tumors like petroclival meningiomas. Thus, it is esteemed as one of the most versatile approaches in the neurosurgical community (12). With the rapid development of neuroendoscopy, the infratentorial space has been recognized as another optimal endoscopic operating area. In 2022, Xie et al. first described EF-SCITA for treatment of petroclival meningiomas (6). We further tested the feasibility of this approach in resection of posterior clinoid meningioma, retroinfundibular craniopharyngioma, as well as trigeminal schwannoma. Under endoscopy, we were surprised to find that the clival region and even the suprasellar area could be clearly exposed through the crural cistern after tentorium incision (13–15). This successful experience motivated us to use the same technique for resection of clival lesions.

This posterolateral approach allows accessing the upper-middle clivus through the petrous-tentorial triangle and crural cistern with the aid of tentorial disconnection. As an important variant of SCITA, supracerebellar transtentorial approach has been used for resection of tumor in the supratentoial areas, such as temporomesial cavernoma, tentorial incisura meningioma, and retroinfundibular craniopharyngioma. Under these circumstances, the tentorium must be opened for establishment of the surgical corridor (13, 14, 16, 17). However, this may not be a necessity for approaching an upper-middle clival lesion. In this case, we cut the tentorium so that the CN IV and SCA could be better exposed, thus facilitating the preservation of these structures while separating the upper pole of the tumor. Apart from tentorium incision, efficient tumor debulking further facilitates the exposure of surrounding neurovascular structures as well as subsequent tumor separation from them. Although it requires working between neurovascular structures for tumor removal in prepontine and interpeduncular cisterns, the manipulation of them was minimal. Compared with the conventional retrosigmoid view, the supracerebellar view supplies the corridor from the medial to cranial nerves, and allows tracing the natural trajectory of CN III-IV, thus reducing the manipulation of neurovascular complex. In addition, it is devoid of cortex retraction seen in the retrosigmoid approach (18). Notably, the wide field-of view and close observation afforded by endoscopy is a precondition for exploiting the natural corridor and protecting critical neurovascular structures. Despite with the long working distance, endoscopy extends our eye to see and our hands to excise this lesion.

Compared the anterior view, this approach allows tumor separation from the brainstem and basilar artery under direct vision, minimizing relevant disastrous morbidities. Postoperative CSF leak is a major complication for midline approaches in clival chordoma resection, which usually requires return for repair or even permanent CSF diversion (19, 20). For EEEA, a series of recent studies reported the incidence of postoperative CSF leak ranging from 3.1% to 22.0%, with the percentage of around 5% mostly reported (19–23). Despite the lack of evidence concerning the incidence of CSF leak in posterior fossa surgery for clival chordoma, a recent meta-analysis indicated that open surgery produced lower rates of CSF leak compared with endoscopic surgery (24).

As mentioned before, a special aspect of this case is that the tumor was separated by a thin, intact membrane structure from the sphenoid sinus. The adoption of this posterolateral route could theoretically preserve this natural barrier. The close observation afforded by endoscopy helps monitoring the degree of tumor resection in the anterior boundary of the tumor under direct vision. Indeed, we succeed in the preservation of this structure and no preventive skull base repair was performed during surgery. This approach also avoids other inherent shortcomings of above-mentioned midline and lateral routes, such as hearing loss, excessive bone drilling, and nasal injury. Another advantage of this technique is the simplified craniotomy procedure, with a small incision that does not obviously affects the appearance of patients. Thus, it might be a promising alternative approach for resection of specific types of clival lesions.

The only study concerning the supracerebellar approach for clival chordoma resection was reported by Goel et al. in 2020 (25). There are several difference between microscopic techniques reported by Goel et al. and endoscopic techniques described herein. First, with a linear light source outside the surgical field, microscopy sometimes fails to render optimal visualization within complex CPA corners. Thus, they have to resort to cerebellar retraction and even sacrifice of the petrosal vein to compensate for the lack of illumination. However, these drawbacks are circumvented by the wide field-of view and close observation afforded by endoscopy. Second, an additional retrosigmoid surgical route was adopted under microscopy for a better exposure and resection of large clival chordomas, which sometimes entails bone drilling of the petrous apex. Here, we succeed in exposing the inferior edge of the tumor located in the midclivus under endoscopy only. We believe that the utilization of angled endoscope and tailor-made microinstruments might minimize the chance of tumor remnants under the condition of a larger clival lesion that penetrates into the mid-clivus. However, the EF-SCITA alone might not be suitable for lesions residing in the lower clivus. Under this condition, an additional retrosigmoid or far-lateral approach might be favored.

Another important discrepancy between Goel’s and our reports lies in the surgical position. Compared with the lateral position, the semi-sitting surgical position in posterior fossa surgery allows for a clean operative field, reduced need for coagulation, and bimanual operations. The surgeon could change the line of sight without the need of rotation of the table. In addition, the venous outflow is promoted, resulting in less venous bleeding (26). Although it seemed to lost favor recently partly due to assumptions of increased risks in venous air embolism (VAE) (27, 28), systematic optimization procedures, including modified sitting positions, meticulous intra-operative monitoring, and proper teamwork, have been revealed to minimize this risk (28–31). The head-up position established here in essence represents a variation of the semi-sitting position in which good cerebellum relaxation can be achieved with “theoretically” lower incidence of VAE. Additionally, its appearance in the lateral position spares complicated preoperative procedures for the semi-siting position. Notably, the endoscopy to some degree improves the shortage in surgical observations that could be compensated by the semi-sitting position under microscopy. Indeed, it turns out to be an efficient, safe, and ergonomic position for endoscopic resection of clival lesions. Nevertheless, we still believe that the combination of the semi-sitting position and EF-SCITA approach may yield further benefits on clinical outcomes of clival lesions, which is a main focus in our future studies.

### 3.3 Limitations of the EF-SCITA approach

Despite the merits mentioned above, we are clearly aware that it is not without flaws. Firstly, as mentioned before, the static endoscopic view lacks dimension and depth of field compared with the dynamic endoscopic technique. The endoscopist may have difficulty in accurately estimating the real distance between the surgical instruments and the visualized structures for handling deep bleeding and sharp dissections. Thus, this technique necessitates a steep learning curve and its real significance relies on the experience of the surgeon. Hopefully, the advent of three dimensional endoscopy may help solve this problem (32). Second, despite the static endoscopic technique, injury can still occur during instrument insertion, potentially without the surgeon’s awareness, especially if the lens is fixed in a deeper plane. Third, the debris produced during surgery easily obscure the lens, which requires frequent flushing of the operative area. Fourth, it is not devoid of brainstem and cerebellum injury. Thus, much attention should be paid to the protection of neurovascular tissue during endoscopic explorations in the supracerebellar space. Fifth, it has inherent shortcomings of suboccipital craniotomy such as injury to the transverse-sigmoid sinus. Last but not least, this case represented resection of a medium-sized lesion. Considering the surgical corridor, it would be an ideal approach for upper clival lesions with posterolateral extension, but may not be enough for complex tumors with extreme lower clivus extensions. In this situation, a far lateral approach or a midline approach may be better.

## 4 Conclusion

To the best of our knowledge, this is the first case report describing the EF-SCITA approach in the treatment of an upper-middle clival chordoma. Overall, this posterolateral approach could achieve radical tumor resection while minimizing neurovascular morbidities. It provides neurosurgeons with a viable alternative to traditional approaches to this rare entity, especially those with their bulk located in the posterior cranial fossa.

## Data Availability

The original contributions presented in the study are included in the article/supplementary material. Further inquiries can be directed to the corresponding authors.

## Footnotes

1
https://www.dropbox.com/scl/fi/ddopuoe4gbuczppi3tazp/Revised-Video-1-Endoscopic-Far-Lateral-Supracerebellar-Infratentorial-Approachfor-Resection-of-Clival-Chordoma.mp4?rlkey=8luzoelmxio2njhbscqefsff2&st=mpsrbqr1&dl=0

2 0:00 This video shows technical specification of endoscopic far-lateral supracerebellar infratentorial approach (EF-SCITA) for resection of upper-middle clival chordoma. The rationale of adoption of this technique was based on our cadaveric study indicating that the clival region could be accessed via lateral supracerebellar infratentorial space under endoscopy, with the aid of tentorium incision. 0:30 In this case, a “head-up” lateral position and Dandy incision was adopted. 0:35 First, the tentorium was opened to facilitate accessing the crural cistern through the petrous-tentorial triangle, during which much attention should be paid to the protection of trochlear nerve. Then, the arachnoid membrane overlying the tumor in the crural cistern was dissected to reveal the tumor. The tumor was found to transgress dural limits into the interpeduncular cistern from upper-middle clivus. 1:06 A small incision was taken on the bulge. The contents mixed with blood component spontaneously bursted out. 1:18 After initial debulking, the tumor was separated from ipsilateral oculomotor nerve and anterior pontomesencephalic segment of superior cerebellar artery (SCA) superiorly. 1:52 Then, the capsule was gently dissected away from the brainstem posteriorly under direct vision, during which attention should be paid to the preservation of the basilar artery. 2:02 Along with tumor debulking, the pituitary stalk, contralateral internal carotid artery (ICA) and oculomotor nerve could be seen. 2:08 The tumor component with interspersed bone pieces was removed using fine microneurosurgical techniques. 2:23 After radical tumor removal, ipsilateral abducens nerve that was displaced laterally could be observed. 2:27 Finally, hemostasis was performed.
